# Modelling the creation of friends and foes groups in small real social networks

**DOI:** 10.1371/journal.pone.0298791

**Published:** 2024-02-27

**Authors:** Alberto García-Rodríguez, Tzipe Govezensky, Gerardo G. Naumis, Rafael A. Barrio

**Affiliations:** 1 Departamento de Sistemas Complejos, Instituto de Física, Universidad Nacional Autónoma de México (UNAM), Coyoacán, CDMX, México; 2 Instituto de Investigaciones en Matemáticas Aplicadas y en Sistemas, Universidad Nacional Autónoma de México (UNAM), Coyoacán, CDMX, México; 3 Instituto de Investigaciones Biomédicas, Universidad Nacional Autónoma de México (UNAM), Coyoacán, CDMX, México; AGH University of Science and Technology Faculty of Physics and Applied Computer Science: Akademia Gorniczo-Hutnicza im Stanislawa Staszica w Krakowie Wydzial Fizyki i Informatyki Stosowanej, POLAND

## Abstract

Although friendship networks have been extensively studied, few models and studies are available to understand the reciprocity of friendship and foes. Here a model is presented to explain the directed friendship and foes network formation observed in experiments of Mexican and Hungarian schools. Within the presented model, each agent has a private opinion and a public one that shares to the group. There are two kinds of interactions between agents. The first kind represent interactions with the neighbors while the other represents the attitude of an agent to the overall public available information. Links between agents evolve as a combination of the public and private information available. Friendship is defined using a fitness function according to the strength of the agent’s bonds, clustering coefficient, betweenness centrality and degree. Enmity is defined as very negative links. The model allows us to reproduce the distribution of mentions for friends and foes observed in the experiments, as well as the topology of the directed networks.

## 1 Introduction

Humankind knows possible ways to solve many of the pressing issues formulated in the United Nations millennium development goals [1]. Yet conflicts play a crucial role to hamper cooperation and the search of rational solutions [2]. Conflicts have been documented since the very dawn of civilization and many theories have been proposed to explain its origin [3–5]. Its study involves many disciplines as psychology, sociology, biology, economy, literature, history, etc. [2, 5, 6]. The arrival of big data and of social networks like twitter or facebook allowed to get further perspectives on the problem. The problem with such kind of networks is that the presence of non-human users makes difficult to compare with a purely human network [7]. Moreover, such social networks do actually lead to different and new forms of organization [8].

Personal relationships are different: there is a physical limitation in the number of close friends and heated enemies [9, 10], usually structured into several layers [11]. This is specially clear in schools as direct interpersonal social interactions are limited in time and space [12]. Mobile networks are useful to explore such close-knit relationships but do not provide information on foes [11, 13].

Several studies have explored the classification of agents as either allies or adversaries using different theoretical frameworks. Game theory has been employed in some works to elucidate the process of distinguishing between friend and foe [14, 15]. Alternatively, a social balance approach has been adopted in many other studies [16–18]. Additionally, some research has focused on understanding how spatial correlations and interactions play a crucial role in determining friendships and enmities [19], while others have exclusively concentrated on the dynamics of friendships [20]. Moreover, investigations have been conducted to analyze the impact of friends and foes on information diffusion [21], strategies to defend friends from bullying [22], and the facilitation of cooperative behavior [23].

Although there are many different explanations and theories on how friends are created [24], few works analyze what are the differences with foe networks. Such questions have been tackled in some experimental studies [25, 26]. For example, in a previous paper some of us performed a study of friends and foes networks in several Mexico city primary and secondary schools [25, 27]. Each student was asked to rank eight friends and foes, although later on only five were used to analyze the data. The protocol in such work was made to assure the secrecy of the data to avoid ethical problems. From the results, a weighted network was obtained. The main finding was that foe networks are much more heterogeneous than the friendship ones, and often a kind of public enemies arise. The study found that below certain age, genders tend to be dissociated, as also found in other studies [12, 15].

A similar experiment was performed in Hungarian high school groups [28]. The main difference with the Mexican experiment was that students classified all others in five categories. As a result, the networks were less dense than the Mexican ones. Yet the conclusions of the Hungary school experiment were somewhat similar to the Mexican one in the sense that friendship and foe networks are different [25, 28].

Our objective is to investigate the reasons that result on the different topological structures between networks of friends and foes. To explain such differences, in this work we go further by proposing a model for the dynamic creation of friends and foes in this small kind of real, interpersonal networks. However, as we will show here, the criteria used to select friends differs from the ones to select foes. The process also depends upon the experimental design and thus the model needs to be adapted according to the questions asked to the students. The dynamical model was directly taken from reference [29] because the dynamical mechanism is quite suitable for this case, as people in principle do not disclose their opinions about others. What is new here, is the procedure of signaling their preferences and keeping the memory of negative interactions. As far as we know no other models in the literature take this approach.

The layout of this work is the following. In section 2 we present the dynamical model and an example of the numerical procedure is to be found in section 2.1.2. Section 3 presents the empirical data. In Section 4 the results from the model re exhibited and compared with the experiments. In Section 5 we discuss the results and finally, in Section 6 some conclusions are given.

## 2 Theoretical model

In order to investigate the mechanisms by which different opinion networks are formed we first have to simulate the dyadic interactions between individuals in order to assess their point of view about a subject. Then we need to evaluate their homophily that allows the identification of best friends and foes.

The model is structured in two parts.

- A dynamical system that describes how the students get acquainted with each other during a certain time.
- A decision making model that represents the way the students designate their friends and enemies.

### 2.1 Dynamical model

For the dynamics of students getting acquainted with each other, our work is based on the model of opinion formation in a society [29–31]. This is an agent-based opinion formation model considering that the agents’ states are defined by a time-dependent variable *x*_*i*_, associated to each agent *i*. This variable is private, in the sense that no other agents know its instantaneous value. The information other agents have is a fraction of its true value.

The social network is represented by an adjacency matrix *A*_*i*,*j*_, in which the weight of the links is positive for friendly relations and negative for enemy relations. Links with zero weight behaves in the same way as if there was no link between individuals.

Initially, *A*_*i*,*j*_ is a matrix generated by making random connections between nodes until a previously defined average degree *k* is reached. At the start of the simulation we assume that there are not enemies and therefore these links have an equal weight 1. The basic dynamical equation for the agent *i* true state *x*_*i*_ can be written as follows

$$\begin{array}{c} \frac{\partial x_{i}\left(t\right)}{\partial t}=f_{s}\left(i,t\right)\left|x_{i}\left(t\right)\right|+\alpha_{i}f_{l}\left(i,t\right), \end{array}$$ (1)

In here the first term on the right hand side represents direct interactions between agent *i* and its neighbours, and the second term represents the long range interactions between agent *i* and the rest of the network. The parameter *α*_*i*_ is a random bounded variable (*α*_*i*_ ∈ [−1, 1]) that represents the attitude of agent *i* to the overall public available information *α*_*i*_, being negative if the agent is inclined to oppose the crowd and positive if the agent simply follows the crowd. Specifically,

$$\begin{array}{c} f_{l}\left(i,t\right)=\sum_{j\in m(\ell =2)}^{m(\ell_{max})}\frac{1}{\ell }y_{j}\left(t\right), \end{array}$$ (2)

where the summation is over all the *m* agents separated from agent *i* by *ℓ* steps and *y*_*j*_(*t*) is the public portion of *x*_*j*_ available to agent *i* at time *t*. For simplicity we assume that the importance of the information decays linearly with the number of steps. Observe that the information that an agent is getting is only about the apparent and public positions of the other agents. The short range interactions simply is,

$$\begin{array}{c} f_{s}\left(i,t\right)=\sum_{j\in m(\ell =1)}^{k_{i}}w_{i,j}\left(t\right), \end{array}$$ (3)

where *w*_*i*,*j*_(*t*) is the instantaneous information that agent *j* passes to agent *i* (not necessarily the same as *w*_*j*,*i*_(*t*)). The summation is over all *k*_*i*_ first neighbours. The absolute value in equation (1) assures that this interaction is homophilic in the sense that is positive when the signs are the same.

It is clear that the sum over the rows of the *W* matrix give the average apparent position of the agent, thus,

$$\begin{array}{c} y_{i}\left(t\right)=\frac{1}{k_{i}}\sum_{j\in m(\ell =1)}^{k_{i}}w_{j,i}\left(t\right), \end{array}$$ (4)

where we have normalised by the degree *k*_*i*_ of the agent, to restrict the *y* value within the interval [-1,+1].

Finally, the amount of information that a given agent is giving to a neighbour is taken to be

$$\begin{array}{c} w_{i,j}\left(t\right)=\tau x_{i}\left(t-1\right)+\left(1-\tau \right)y_{j}\left(t-1\right), \end{array}$$ (5)

where *τ* is a parameter ranging from zero to one that measures how much an agent is prepared to go public. In principle this should differ from agent to agent, but we take it equal for all agents, so it could be used as characterizing the mean value of the openness for the whole network.

#### 2.1.1 Evolution of links

The strength of the links represents the degree of acquaintance between two individuals. Therefore, as a consequence of the interactions the weights vary in time according to,

$$\begin{array}{c} \frac{\partial A_{ij}}{\partial t}=DT_{ij}\left(t\right), \end{array}$$ (6)

where the constant *D* sets the time scale for the linear growth of the link. *T*_*ij*_(*t*) should be a function of all the variables involved in each link, namely (*x*_*i*_, *y*_*i*_) and (*x*_*j*_, *y*_*j*_). We take it to be

$$\begin{array}{c} T_{ij}\left(t\right)=\left|\left(x_{i}\left(t\right)+x_{j}\left(t\right)\right)/2+3\left(y_{i}\left(t\right)+y_{j}\left(t\right)\right)/2\right|-1, \end{array}$$ (7)

which takes values between -1 and 3. Observe that if the disagreement between agents having opposite values of their variables results in a negative value of *T* and a decrease of the weight of the link, which eventually becomes negative. We interpret positive links as friendly relations and enemies have negative links.

The network is modified every 50 iterations to spot the interactions that are not working. The number of links that become negative are detected, and one creates he same number of positive new links by choosing the individuals with lower degree, and if this is not possible, one chooses an agent at random. However, the original links still evolve with the dynamics, and some of them develop positively, but some others become more negative under the new network dynamics. These latter ones are used to detect the real enemies.

#### 2.1.2 Numerical results of the dynamical model

In order to show the kind of networks that are formed with this model, we performed extensive numerical calculations using a simple Euler method with a time step small enough (*dt* = 0.002) to avoid numerical artifacts. The parameter *α*_*i*_ was taken from a uniform random distribution in the interval [-1,1] and the adjacency matrix was a random matrix with average degree 〈*k*〉 and initially with weights equal to one. The initial values for the state variables *x*_*i*_ and *y*_*i*_ are assigned randomly from a uniform distribution [-1,1].

**Fig 1:**
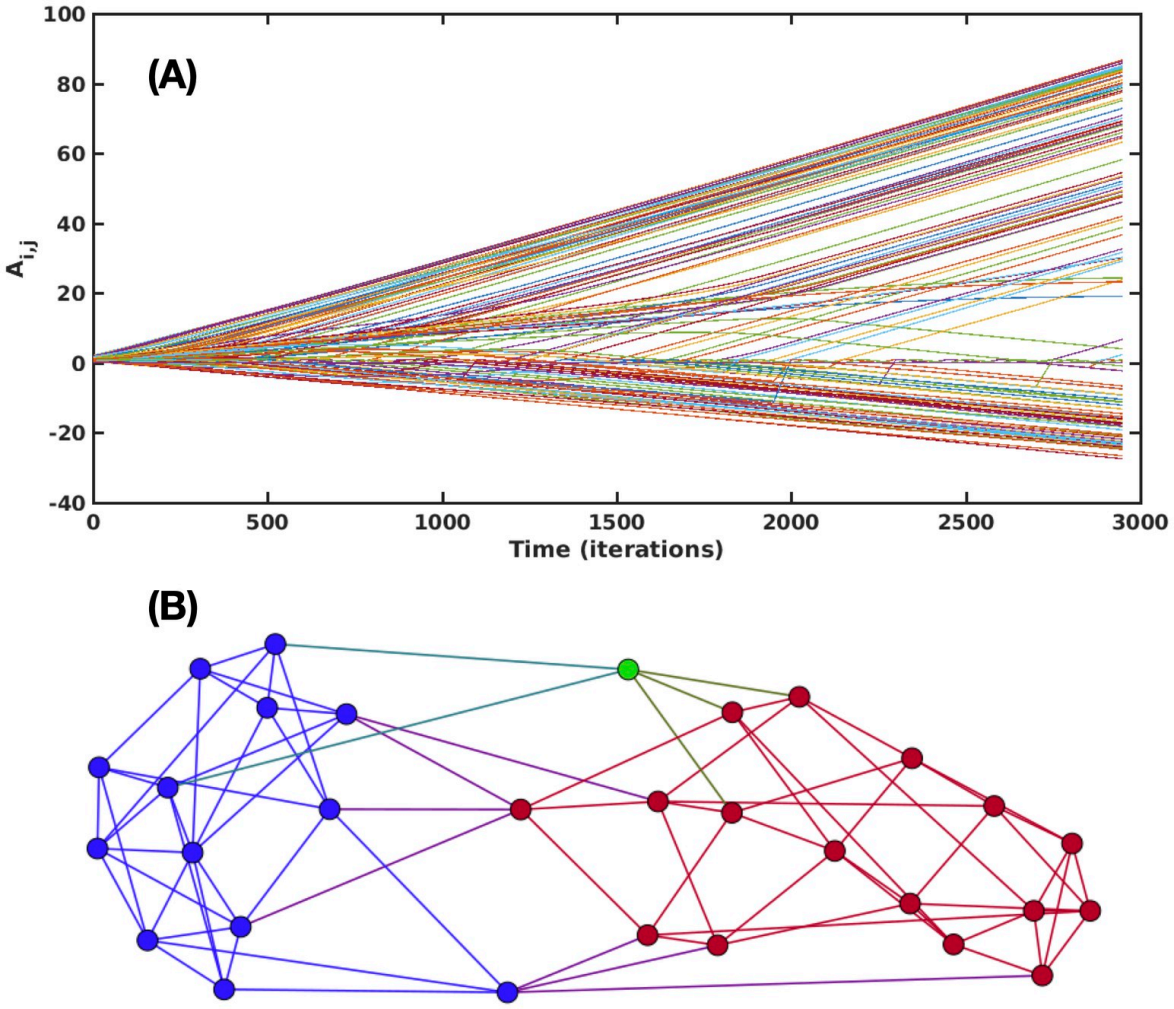
Time history links and network results from the dynamical model. (A) Time history of the weights of the links, according to Eq 6. (B) Final network of positive links decorated with the values obtained from the dynamical model. The blue nodes have reached the minimal values of *x* = *y* = −1. The red nodes have *x* = *y* = 1 and the green node did not reached these extreme values.

In Fig 1(A) we show a typical time evolution of the adjacency matrix. Observe that not all lines are straight, reflecting the complexity of the interactions. Positive links reflect friendly bonds and enemies are detected by the links that become very negative despite rewiring Fig 1(B) shows a typical network obtained numerically from this model, taking *τ* = 0.8 and an average degree of 〈*k*〉 = 6. The blue circles are nodes with final *x* = −1, the red circles are nodes with final *x* = 1, and the green ones are agents that ended up in a locked intermediate value of their state variable.

### 2.2 The process of friends and enemies designation

After the dynamical model is computed, we simulate the process by which an individual is seen as friend or foe by others. Designating friends and enemies depends on several features of the relationship and it also reflects the instructions they are given in order to enlist friends and foes.

Although the sign and weight of the links determine friend or foe relations, the way the students designate them also depends on other criteria such as popularity and friendliness. To take this into account we define a fitting function for each agent, considering not only the strength of the agent’s bonds (∑(*A*)), but also their clustering coefficient (*Cl*), betweenness centrality (*bt*), and degree (*k*), in equal footing,

$$\begin{array}{c} fit\left(i\right)=(bt\left(i\right)+Cl\left(i\right)+k\left(i\right)+|\sum_{j=2}^{N}A\left(i,j\right)|)/4, \end{array}$$ (8)

where the numerical factor is a normalizing constant. This function takes into account with equal footing all network indicators that depend on a single agent.

A priori, it is not clear how these features contribute to the definition of homophily. The experiments have no information about this issue. Therefore, we tried calculations with different combinations of the four properties in Eq 8 to discern which ones were more important to give reasonable results. We found that when one omits one, two or three of them the predictions of the calculation cannot be used to fit the data in a satisfactory way. The best results were obtained when we used Eq 8 with equal weights (see S1 Appendix). In the results section below we show only the best fit, leaving the analysis of the homophily factors to future work.

Assuming that friendship and enmity are two extremes of a homophily scale we calculated: Δ_*f*_(*i*, *j*) = |*fit*(*i*) − *fit*(*j*)| and used the smallest values of Δ_*f*_(*i*, *j*) to designate friends, and the largest values to designate enemies. In Section 4.1 we will explain how the voting procedure is adapted to the constrictions imposed by different experiments.

## 3 Data description

We are now applying our theoretical model to real social networks and comparing it with publicly available and published data from experiments made by other groups: one conducted in Mexico [32] and the other in Hungary [28]. Taking part in the experiments was voluntary, and parents’ consent was obtained. Confidentiality was very important, and students were assured that their answers would be used exclusively for research purposes. For both studies, the response variables were the sum of the number of mentions received by each student, as friend or foe, listed by the entire class. Below we describe the protocols followed by such experiments, as they will be used for comparison with our results.

### 3.1 Mexican data

The experiment was performed in several schools in Mexico City [32]. Data from 37 school classrooms and 4 groups of teachers were collected. Participant’s ages ranged from 10 to 40 years. Group sizes varied from 9 to 36 individuals.

Students were asked to indicate five classmates which she/he likes most (we classify them as friends) and five classmates which she/he dislikes (foes).

**Fig 2:**
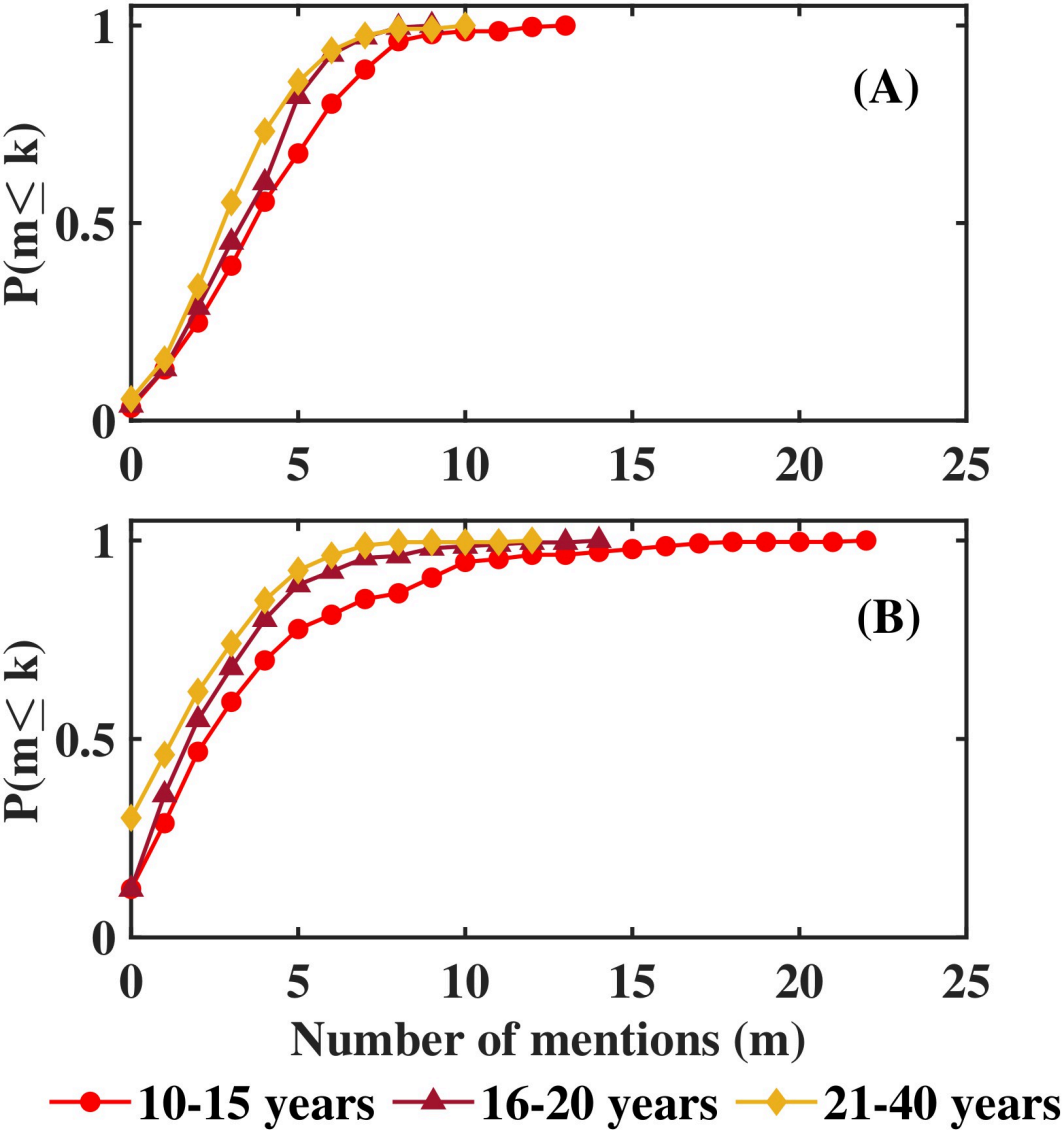
Distribution of friends/foes.

Since ages varied greatly in this study we analyzed the number of mentions classifying data in 3 categories: 10 to 15 years old (11 classrooms), 16 to 20 years old (14 classrooms), and 21 to 40 years old (16 classrooms). Fig 2 shows the cumulative distribution plots obtained for mentions for friends (Fig 2A) and foes (Fig 2B) for the 3 age categories. Notice that as students get older they mentioned less friends, and are even more reluctant to mention enemies.

Distribution of the summed numbers of friends/foes that each student has as listed by the entire class (x axis), for friends (A) and foes (B) in Mexican data separated according to age. As people get older they are more selective when mentioning friends; more so when mentioning foes, even though they were asked to mention 5 foes, the average number of foes mentions decreased from 3.9 to 2.2. The y axis denotes the relative accumulated frequency. Notice that only about 20% of the students has more than 5 friends; although the maximum number of mentions as a foe that a student received was 23, only about 10% were signalled as enemy by more than 8 classmates.

Since distributions were different according to age, we decided to work with data for students between 10 and 15 years old. In this group, classrooms had on average 25.3 students; the average mentions for friends was 4.4 an for foes was 3.9. Therefore, in order to simulate this experiment we used networks of 25 agents and considered that they were asked to mentions 4 friends and 4 foes.

### 3.2 Hungarian data

The second experiment was performed in Hungary (RECENS high school dataset [28]). Data were collected in 43 first grade high school classes from 7 schools. Students’ average age was 15.9 years. Group sizes ranged from 17 to 39 students, averaging 32 students. Therefore, we used networks of 30 agents in our simulations. Data were collected longitudinally, we used only the data from “wave 2”, after students knew each other for 6 months.

Data were collected by applying questionnaires where students were asked, among other things, to rank all their classmates: “−2” for enemy, “−1” for dislike, “0” for neutral, “1” for like and “2” for friend. We used only the two extreme values: “−2” as enemies and “2” as friends.

## 4 Results

This section is divided in two subsections. In the first one we present the criteria to designate friends and foes, and explain how to adapt the voting procedure to the requirements of each experiment. In the second subsection, a comparison between the model simulations and experimental results is presented.

### 4.1 Designating friends and foes

The dynamical model simulates daily interactions between students getting to know each other. Besides opinion exchange interactions there are other factors influencing the choice of friends and foes, such as popularity and friendliness.

Therefore, our first hypothesis was to consider that friends and enemies are selected using the same criteria defined by a homophily scale Δ_*f*_(*i*, *j*). When using this method, the simulations approximated fairly well the data for friends, but not for foes but not quite for foes.

These results led us to conclude that the criteria used to vote for a classmate as a friend is essentially different from the ones used to consider a mate as an enemy. Indeed, friends are chosen taking into account the strength of positive bonds developed during daily interactions, and also popularity and friendliness, Δ_*f*_(*i*, *j*) is appropriate for this task. In contrast, enmity depends on personal experiences that not only creates negative bonds but causes its rupture. Thus, to simulate enmities we decided to use the most negative values of *A*_*ij*_.

How small should Δ_*f*_(*i*, *j*) be to consider agent *i* as a friend, or how negative should *A*_*ij*_ be to designate agent *j* as an enemy, depends on the instructions given to the students. It is in this sense that we need to adapt the voting mechanism for each particular experimental situation.

The Mexican experiment was simulated by choosing the 4 smallest values of Δ_*f*_(*i*, *j*) (i.e. greatest homophile) as friends of agent *i*, and the 4 most negative values of *A*_*ij*_ as enemies of agent *i*. Since many agents did not have 4 negative bonds, all negative bonds of agent *i* were designated as foes, and to complete to 4 foes, as requested in the experiment, the required number of highest values of Δ_*f*_(*i*, *j*) were added to the list.

In order to simulate the Hungarian experiment, where each student ranked his classmates according with his own criterion, we defined two parameters (*β*_1_ and *β*_2_) used as thresholds. Values of Δ_*f*_(*i*, *j*) < *β*_1_(*i*) were considered as agent’s *i* friends and links having *A*_*ij*_ < *β*_2_(*i*) were designated as foes. To select the values of *β*_1_ and *β*_2_ the distributions of Δ_*f*_(*i*, *j*) and *A*_*ij*_ based on 40 simulations were obtained. *β*_1_ was fixed at the 12*th* percentile of the first distribution, *β*_2_ was made equal to the 30*th* percentile of the second distribution. To reflect the fact that each student has his personal threshold when designating friends and foes, *β*_1_ and *β*_2_ were calculated for each agent: *β*_1_(*i*) = *β*_1_ + *ϵ*(*i*) and *β*_2_(*i*) = *β*_2_ + *ϵ*(*i*) where *ϵ*(*i*) is a random number taken from a uniform distribution in the interval [−0.5, 0.5].

### 4.2 Comparison between data and model

**Fig 3:**
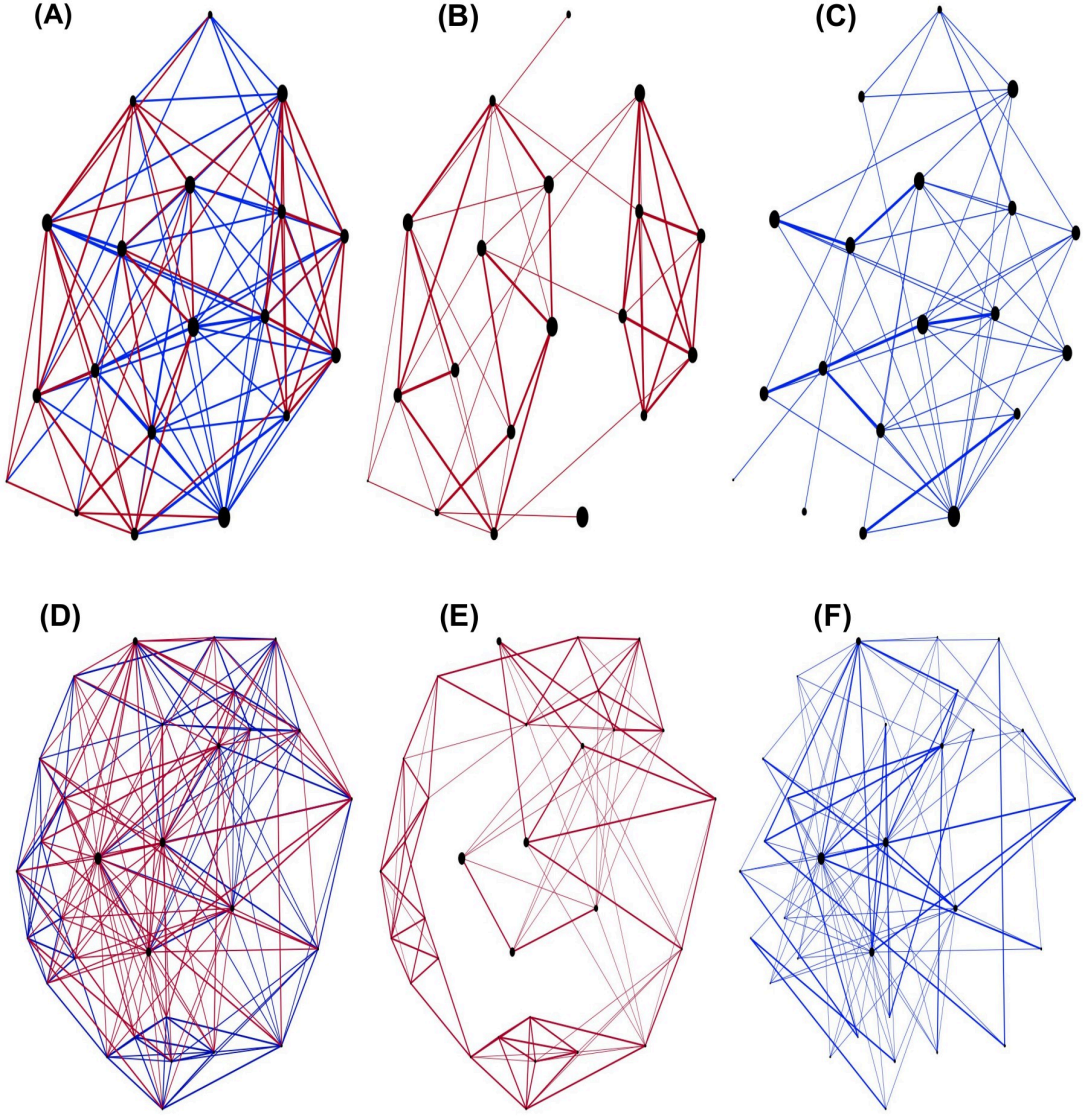
Mexican networks. (A,B and C) Networks corresponding to a group from the experiment in Mexican schools. (D,E and F) Network resulting from a simulation of the Mexican schools. Red links belong to friends relationship and blue links to foes relationship (thick links are reciprocal links).

**Fig 4:**
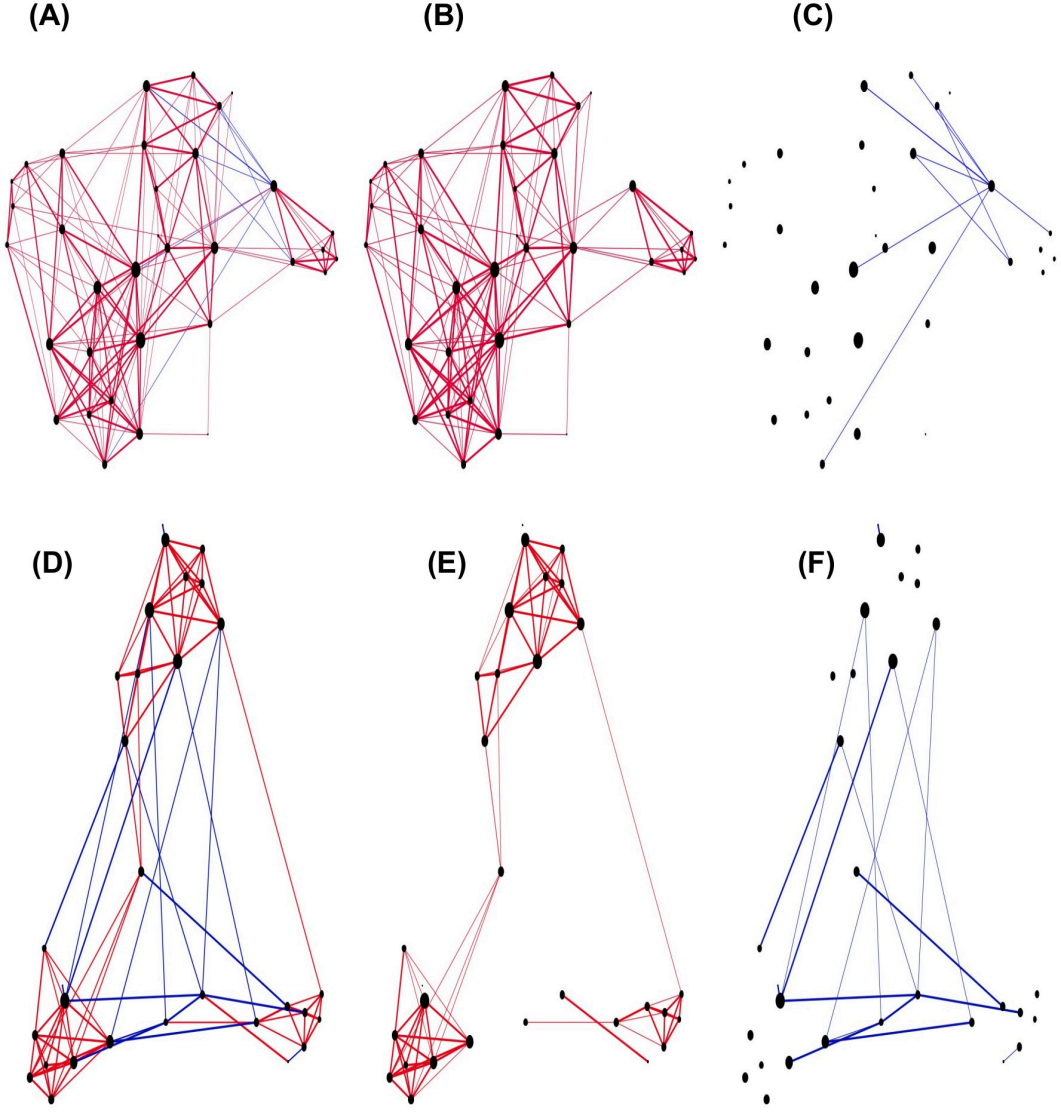
Hungarian networks. (A,B and C) An example of a real network from Hungarian schools. (D,E and F) Example network from a simulation of the Hungarian schools. Red links belong to friends relationship and blue links to foes relationship (thick links are reciprocal links).

A characteristic of friends and foes networks in Mexican and Hungarian experiments is their asymmetry. It shows that mentions for either friends or foes are not necessarily reciprocal. Our simulations reproduce these results (see Figs 3 and 4). The model also shows that in the Mexican experiment there are few individuals signalled by many people as enemies, but not so in the Hungarian experiment. This is a consequence of the fact that Mexicans were asked to name 5 enemies, while the Hungarians were not, and they only mentioned one person as enemy on average. The empirical observation that nodes with highest degrees, in the case of Mexican data, have in general mainly enemies but also some friends; and in the case of Hungarian data they have, in general, many friends and also few foes.

Observed friendship networks, in both experiments, are constructed by groups of friends interconnected by students who mentioned members of other groups, creating links between groups; whereas foes networks do not have this structure. In the Mexican experiment, highly connected networks of foes are observed without a specific structure; for Hungarian data, many students have no mentions as foes, in some cases there are no real nets, and when nets are formed, they are very sparse, without structure. Our model reproduces these characteristics. Fig 3 shows friends-foes networks obtained for one class of the Mexican experiment (A, B, C), and for one simulation where 4 friends and 4 foes were chosen by each agent (D, E, F). In Fig 4(A)–4(C), similar graphs are shown for the Hungarian experiment and in Fig 4(D)–4(F) we show a simulation using agent’s threshold for choosing friends and enemies.

Although the quantitative similarity between the experimental and the theoretical networks is not achieved, we can notice an important difference between friendship and enmity networks, namely that friends tend to be in tight clusters while enemies are more isolated, and that the voting procedure has a great influence on the results. These features could be quantitatively compared by looking at the cumulative distributions.

**Fig 5:**
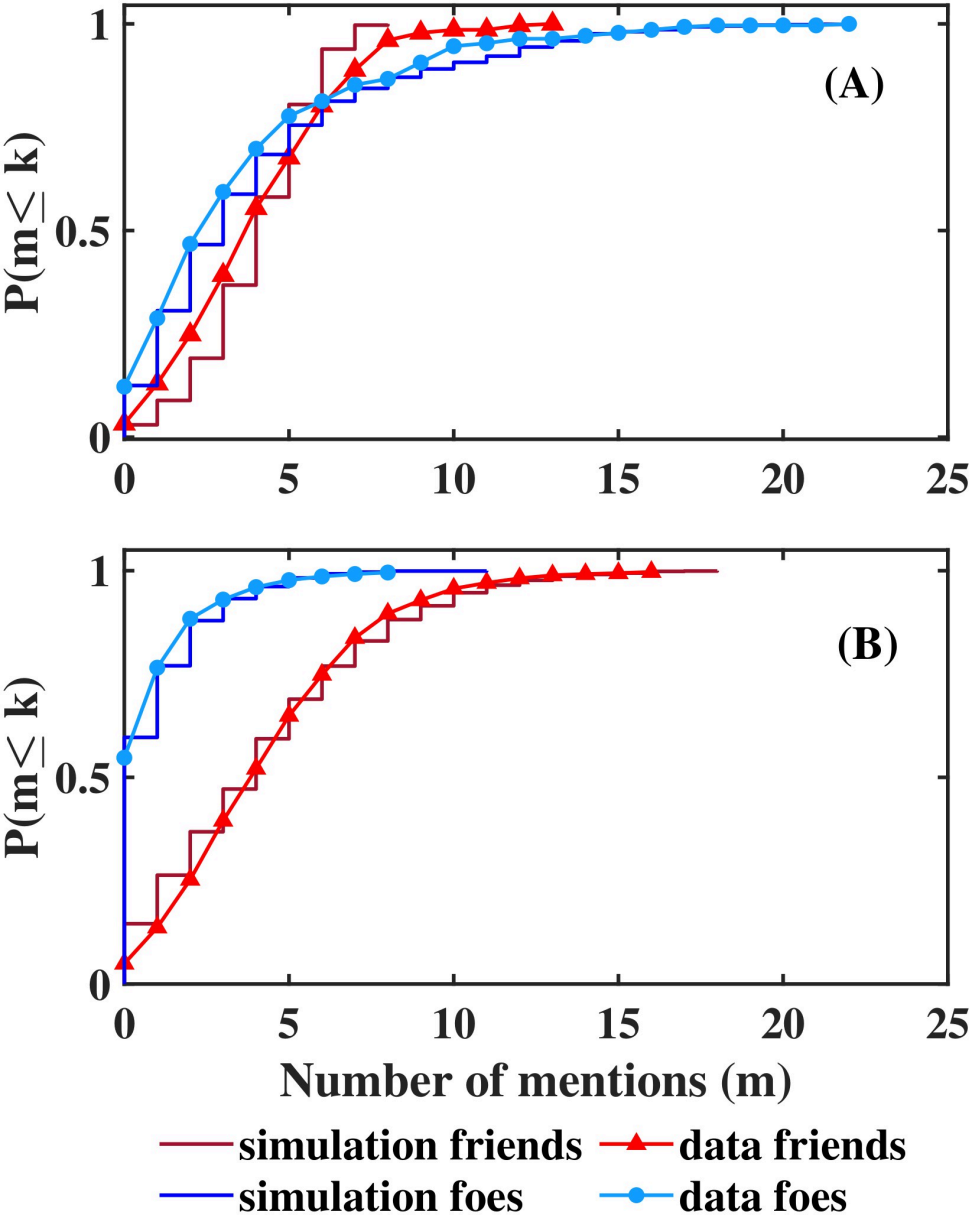
Distributions of number of mentions friends/foes that each student received. Distributions (integrated density function) of number of mentions (the summed numbers of friends/foes that each student has as listed by the entire class (x axis)), relative accumulated frequency (y axis). Only the data for the individuals aged from 10 to 15 in Fig 2 are shown. Simulation results are plotted as stair graphs, data distributions are plotted as continuous lines (A) Mexican data (B) Hungarian data. Observe that about 50% of the the Hungarian students received no mentions as enemies.

Aiming to analyze the experiments as a whole we calculated the cumulative distributions of the number of mentions as friend, and number of mentions as foe for the two experiments separately, including all the classrooms reported in each one of them. To calculate the results obtained from the model, 40 realizations were used. Simulation’s results were similar to Mexican experimental data, and more so for Hungarian data (Fig 5).

**Fig 6:**
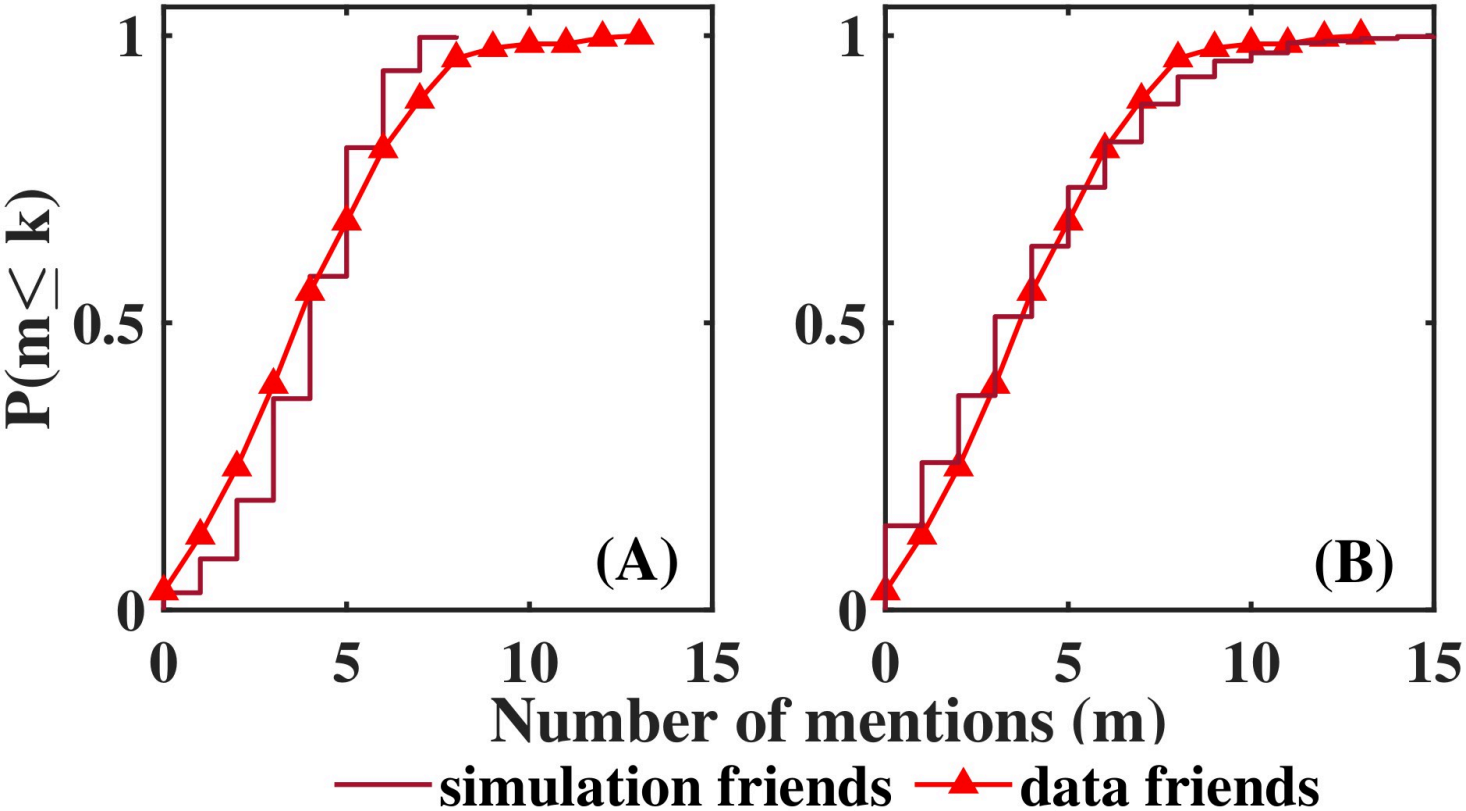
Distributions of number of mentions only friends (simulation results). Distributions (integrated density function) of number of mentions. Simulation results are depicted as stair graphs, data distributions are shown as continuous lines (A) Simulating choosing 4 friends (B) Simulation done as for Hungarian data. The y axis denotes the relative accumulated frequency.

Interestingly, the distribution for friends obtained in both experiments were very similar. Moreover, Mexican data were better adjusted when the simulation used a threshold as for Hungarian students, than when choosing the 4 highest values of Δ_*f*_(*i*, *j*), as shown in Fig 6. This result is probably because Mexican students were asked to mention 5 friends *a fortiori*, and Hungarian students mentioned 4.7 friends on average, coinciding with friendship networks described by Dunbar (Dunbar’s number [33]).

In contrast, foe’s distributions differ between the two experiments, when students were asked to rank their classmates in five categories (Hungarian case), few of them were classified as foes, 55% of the students did not received any mention as enemy, and the maximum number of mentions as enemy was 8, smaller than the maximum number of mentions as a friend observed (19 mentions). When students were asked to mention 5 foes (Mexican case), only 18% of the students did not have any mention as a foe, and the maximum number of mentions observed was greater for foes (22 mentions) than for friends (13 mentions).

## 5 Discussion

Designating friends involves personal opinion, and also friendliness and social references such as popularity. Enmity results from personal interactions leading to a rupture between two people. Friendship and enmity are not two extremes of the same homophily scale, each has its own motivations. Friendship networks are structured into sub-networks, whereas enemy networks are more defuse.

Comparing Mexican and Hungarian experiments let us understand that when students are allowed to mention friends or foes without any restrictions, they use their personal choices. In the case of friends, the fact that we adjusted better Mexican data simulating personal criterion by means of a threshold, points to the fact that students were not affected by the restriction of mentioning 5 friends. But in the case of foes, Hungarian students mentioned few enemies, in contrast, Mexican students used their own point of view, and then, the collective opinion came into play; the universally hated person or *“public enemy number one”* only appears through this mechanism.

Based on survey and simulation results, we think that the special constraint of forcing participants to vote for a specified number of subjects (be friends or foes) may create a false illusion about people’s popularity (be good, bad or both). The latter has implications for example in voting processes, when asking people to nominate a certain number of candidates for a specific position and then counting votes for each candidate, some of them may receive more votes than the number they would have received in a direct selection among a list of candidates.

Our model reproduces the observation that well known or popular persons (agents with high degree), have positive as well as negative links. For Mexican students, usually popular students had more enemies than friends as a result of an exaggerated number of foes caused by having to select 5 enemies; the opposite (more friends than foes) was obtained for Hungarian popular classmates.

By including heterogeneity of criteria among the agents we were able to model accurately the non-reciprocal cases. The lack of reciprocity in naming friends and enemies is due to the fact that individual criteria are different.

## 6 Conclusions

The model presented here captures fairly well the main factors in play for the selection of friends and foes as the resulting number of mentions distribution reproduces well the experimental results. Also, it is worth emphasising that friendship networks are structured in small groups while enmity networks are not. The models also reproduces the asymmetry observed in friend and foes networks.

The main conclusion of this study is that criteria of friend selection includes personal opinion and social perception of the student while enmities result from personal frictions. Another conclusion is that we are more prone to make our friendships known by others while we are reluctant to make public our enmities.

In addition, using our model we were able to infer that different individual criteria cause the lack of reciprocity in friends and foes networks; to explain the emergence of *“public enemy number one”*; and to reproduce the fact that people who have many links have both, positive and negative links.

The structure of networks of friends in small groups do not seem to be the same as those studied in large networks as no large “hubs”, power laws, or small world structure are observed.

We found that in these networks there are small groups with few connections between groups, maybe reflecting a general structure of human societies.

## Supporting information

S1 Appendix (PDF)
