# Supplementary material for: Modelling the creation of friends and foes groups in small real social networks
Source: PLoS One. 2024 Feb 27;19(2):e0298791. doi: 10.1371/journal.pone.0298791 (PMC10898769; doi:10.1371/journal.pone.0298791)
Supplement: S1 Appendix — (PDF) [file pone.0298791.s001.pdf]

8 Appendix

The general dynamical model is versatile enough to handle various situations, depending on the scale of the network. In this study, our focus is on analyzing small, tight-knit networks in classrooms. Therefore, the mechanism for designing friendships depends on the specific cultural and social relations within these groups.

Surely we could assume that the individuals properties should be taken into account, but there is no information in the data to discern which ones are more important to define hommophily amongst the agents. In these circumstances one should take into account all the node properties of the network as stated equation 8. In Fig 1 . we show the results of the prediction of calculations with different combinations of the quantities used. Observe that the best fit the data is obtained when all four properties contribute equally in the fitness function.

Fig 1. Cumulative distribution of different fitting functions Cumulative distribution obtained using different fitting functions compared with Hungarian data for friends. Step graphs correspond to simulations, continuous red line represent the data

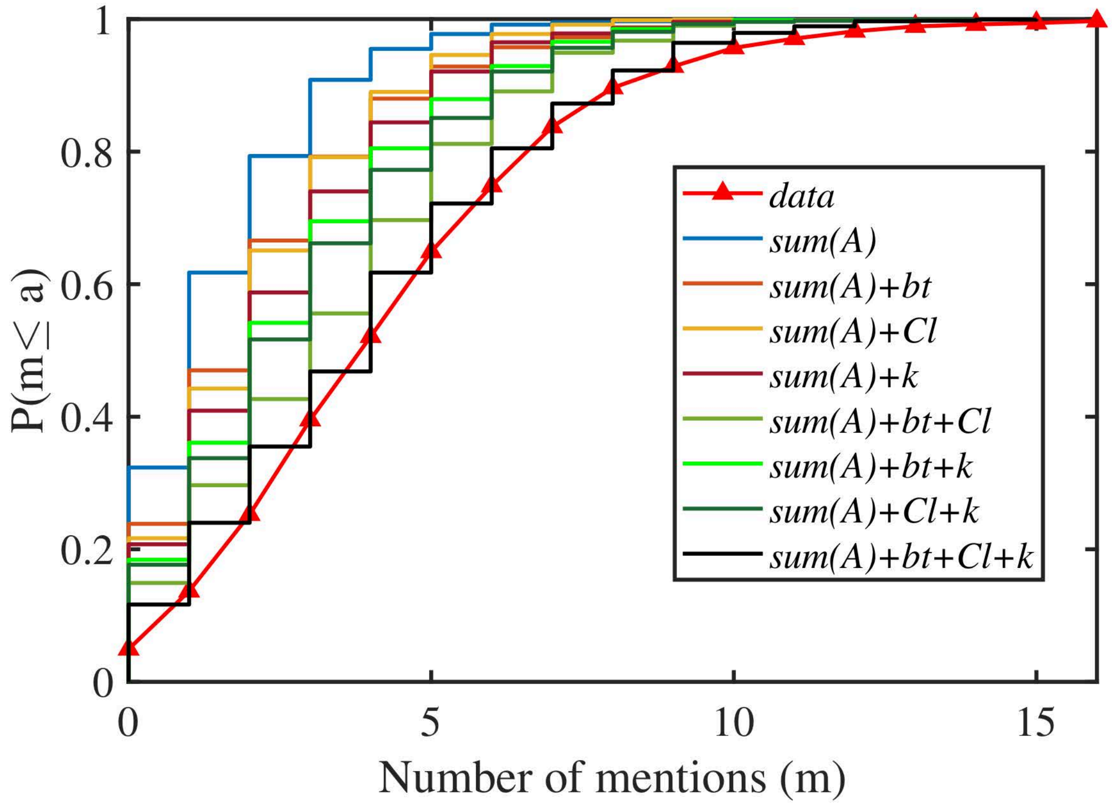

In the figure we show the results of 40 simulations to that render the networks obtained after the dynamical process ($N = 30$, $\bar{k} = 6$). Eight different fitting functions were applied to these networks.
